# Antihypertensive and antioxidant effects of food‐derived bioactive peptides in spontaneously hypertensive rats

**DOI:** 10.1002/fsn3.4404

**Published:** 2024-08-25

**Authors:** Yuefan Zhou, Yixin Xu, Tongguan Tian, Yanping Xu

**Affiliations:** ^1^ Nourse Centre for Pet Nutrition Wuhu China

**Keywords:** antioxidant, blood pressure, inflammation, peptides, spontaneously hypertensive rats

## Abstract

Hypertension significantly impacts the survival and quality of life of animals, often leading to chronic kidney failure. Current clinical drugs used to manage hypertension carry the risk of causing adverse reactions. In contrast, certain natural peptides have demonstrated the ability to safely reduce blood pressure by inhibiting the production of angiotensin. We administered four biologically active peptide solutions to spontaneously hypertensive rats: derived from corn, wheat, egg white, and soybean. The efficacy of these peptides in reducing blood pressure was assessed through regular measurements of systolic pressure. Additionally, we analyzed levels of angiotensin‐converting enzyme and angiotensin 2 using immunohistochemistry and ELISA in vivo. The indicators of oxidative stress and inflammation in hypertensive rats were evaluated using qRT‐PCR and ELISA, respectively. Both wheat (from 182.5 ± 12.26 mmHg at day 0 to 168.86 ± 5.86 mmHg at day 20, *p* = .0435) and soybean (from 189 ± 2.19 mmHg at day 0 to 178.25 ± 5.14 mmHg at day 20, *p* = .0017) notably lowered systolic blood pressure compared to their starting systolic blood pressures in spontaneously hypertensive rats. Both wheat and soybean peptides significantly reduced plasma ANG II levels, akin to captopril’s effect. Wheat peptides additionally exhibited antioxidant properties. Only the corn peptide showed a significant increase in transcript levels of the proinflammatory factors IL‐6 and TNF‐α. At the protein level, all four kinds of peptides significantly elevated IL‐6 levels while inhibiting TNF‐α secretion. This study demonstrates that wheat peptides and soybean peptides administered as dietary supplements exhibit significant hypotensive and antioxidant effects.

## Graphical abstract

Wheat peptides and soybean peptides significantly reduce systolic blood pressure in spontaneously hypertensive rats by affecting the renin‐angiotensin systems. We also observed that wheat peptide appeared to significantly alleviate oxidative stress and inflammatory response.

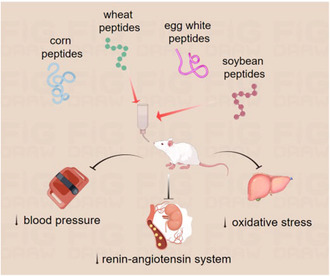

## 1 INTRODUCTION

Hypertension is an important cause of death worldwide. According to statistics, nearly 1.4 billion adults suffer from hypertension (>140/90 mmHg), accounting for 20% of women and 25% of men, and its incidence increases with age (Collaboration, 2021). Hypertension is also a huge medical burden as it is a major risk for stroke, myocardial infarction, kidney failure, and heart failure (Hall et al., 2021). Many studies report that hypertension is an inflammatory process that involves the migration and accumulation of innate and adaptive immune cells into the interstitium of affected tissues, capable of releasing cytokines and promoting oxidative stress (Agita & Alsagaff, 2017; Griendling et al., 2021). First‐line therapeutic agents for hypertension include thiazide diuretics, angiotensin‐converting enzyme inhibitors or angiotensin‐receptor antagonists, and calcium‐channel blockers, which are effective in lowering systolic pressure in patients (Carey et al., 2022). However, in order to enhance the antihypertensive effect, several first‐line antihypertensive drugs are typically combined in clinical practice (Boutouyrie et al., 2021). Alternatively, in order to protect target organs and reduce the incidence of complications, cardiovascular drugs such as statins are used in combination (Messerli et al., 2018).

Bioactive peptides derived from natural products can also play a significant role in reducing blood pressure. Modern nutrition research has found that proteins are not mainly absorbed as widely believed in the form of single amino acids after the enzymes of the digestive tract, but more in the form of polypeptides (Bennett, 1973; Moughan et al., 2014). Because of the intestinal wall, small peptides are less osmotic than free amino acids, reducing the osmotic pressure and improving the absorption efficiency (Daniel, 1969). Therefore, polypeptides are absorbed more and faster than free amino acids. It has been shown that some natural peptides from plants or animals still have biological activities after separation and purification, such as antioxidant effects (Chatterjee et al., 2018), regulation of lipid metabolism (Qiao et al., 2021), protective effects on alcohol‐induced liver damage (Yamaguchi et al., 1996), and promotion of wound healing (Sui et al., 2020). The purified peptides from soybean, corn, egg white, and wheat could inhibit the activity of angiotensin‐converting enzyme II (ACE II) (Mason et al., 2020; Yu et al., 2017). However, differences in purity and peptide sequence make the role of these natural peptides as nutrients added to the diet in lowering blood pressure unclear. In this study, four kinds of purified natural peptides were selected to feed spontaneously hypertensive rats (SHR) in a free‐feeding way by preparing aqueous solution and to explore their alleviating effects on hypertension symptoms as supplementary nutrients.

In normal physiological state, oxidation and antioxidant system maintain homeostasis (Sies, 2021). Oxidative stress protects the body through the regulation of inflammation. When oxidative stress overreacts, excessive accumulation of free radicals causes the antioxidant capacity insufficient (Sies et al., 2017). The release of oxidized substances triggers an attack on cell protein, lipid, and nucleic acid. Therefore, the oxidative damage leads to the occurrence of a variety of diseases, such as vascular endothelial dysfunction, blood vessel function abnormality, and hypertension (Sies, 2023). Studies have confirmed that polypeptides effectively reduce blood glucose and lipid levels, improve the activity of antioxidant enzymes, and inhibit oxidative stress damage in rats (Arulselvan et al., 2016; Rapa et al., 2019). To explore the relationship between oxidation and hypertension, we detected malondialdehyde (MDA), oxidized glutathione disulfide (GSSG), and reduced glutathione (GSH) levels in SHR model which were fed with natural peptides (Elias et al., 2008).

## 2 MATERIALS AND METHODS

### 2.1 Peptides

Corn peptides were composed of a mixture of peptides, which consist of CPP1 (<1 kDa), CPP2 (<3 kDa), and CPP3 (<10 kDa), and purified with corn gluten meal using the method from Xiao Yu (X. Yu et al., 2023). The wheat gluten protein was hydrolyzed by neutrase for 240 min, then the enzyme was inactivated in boiling water bath for 10 min. After centrifugation at 4000 r/min for 10 min, the supernatant, namely the enzymatic solution, was collected, concentrated, and lyophilized to obtain the polypeptide powder. Wheat peptides with molecular weight less than 1 kDa include a variety of pentapeptides and hexapeptides (Wang et al., 2022). The preparation of soybean polypeptide was based on the method provided by Fengguang Pan (Pan et al., 2021). The obtained soybean peptides were composed of a mixture of peptides with molecular weights between 150 Da and 1 kDa. Egg white peptides were obtained by whey protein isolate according to the study by Lu Liu (Liu et al., 2019). Corn peptide, wheat peptide, egg white peptide, and soybean peptide were all provided by the Nourse Centre for Pet Nutrition. Captopril (T1462) was purchased from TargetMol Chemicals Inc.

### 2.2 Animals and experimental design

Seven‐week‐old male SHR rats were selected (animal license number: SCXK (Beijing) 2021‐0006) and divided into six groups: control group (CON, *n* = 6), captopril group (*n* = 6), corn peptide group (CP, *n* = 6), wheat peptide group (WP, *n* = 6), egg white peptide group (EP, *n* = 6), and soybean peptide group (SP, *n* = 6). After a week of acclimatization, the experimental treatment was carried out. The rats in the positive control group were given captopril 20 mg/kg/day by gavage every day, and the rats in the peptide treatment group were given the corresponding high‐concentration peptide solution 4000 mg/kg/day, and the rats were allowed to intake ad libitum. The rats were fed in a stable environment, with room temperature of 25°C, light cycle (12 h:12 h light and dark), humidity of 40%–50%, and free access to water and sterilized feed.

### 2.3 Measurement of blood pressure in rats

To accurately obtain blood pressure, BP‐2010A noninvasive sphygmomanometer was used with tail pressure method. Fix the body of the rat with an appropriate type of mesh bag, put the sensor on the root of the tail of the rat, and monitor the blood flow of the tail artery of the rat through the sensor when the rat is in a stable state. Noninvasive blood pressure measurements were performed on the day prior to peptide administration, as well as on days 4, 12, and 20 following peptide administration. Each session included recording systolic, diastolic, and mean blood pressure, along with heart rate for each rat. Each measurement was repeated three times with intervals of at least 1 minute, and the mean value of each parameter was utilized for statistical analysis.

### 2.4 Preparation of rat plasma and determination of angiotensin II

The rats were euthanized and blood was collected from the abdominal aorta and placed in tubes containing the anticoagulant sodium heparin. The tubes were allowed to stand at room temperature for 20 min and then centrifuged at 3000 rpm for 30 min. The resulting supernatant was collected as plasma, which was subsequently stored at −80°C. The rat ANG II ELISA kit was used to detect the content of ANG II (ng/L) in the sample according to the kit instructions. ELISA kit was purchased from Nanjing Jiancheng Bioengineering Institute.

### 2.5 Preparation of kidney homogenate and determination of inflammatory factors

Cut unilateral kidneys and put the specimen on ice. Weigh 0.05 g tissue and add 0.5 mL RIPA lysis buffer. Grind the tissue with SIENTZ‐48 high‐throughput tissue grinder for 120 s at 60 Hz, and centrifuge at 4°C and 5000 rpm for 10 min. The supernatant was subpackaged for testing. Total protein (g/L) was quantified by BCA assay. The levels of IL‐6, IL‐1β, and TNF‐α (ng/L) in rat kidneys were detected by ELISA kit purchased from Nanjing Jiancheng Bioengineering Institute. The relative level of these inflammatory factors (ng/g) was calculated with the total protein concentration. Extract tissue RNA by Trizol, and the mRNA levels of *Nrf2*, *Il‐2*, *Il‐6*, and *Tnf‐α* in livers were measured by qPCR. Primer sequences are as follows:

Rat Nrf2‐F CGAGATATACGCAGGAGAGGTAAGA.

Rat Nrf2‐R GCTCGACAATGTTCTCCAGCTT.

Rat Tnf‐α‐F CTACTGAACTTCGGGGTGATCGGTC.

Rat Tnf‐α‐R CTGGTATGAAGTGGCAAATCGGCT.

Rat Il‐2‐F TGGAGCAGCTGTTGCTGGAC.

Rat Il‐2‐R TGGCTCATCATCGAATTGGCACT.

Rat Il‐6‐F CCAGTATATACCACTTCACAAGTCGGA.

Rat Il‐6‐R CAAGATGAGTTGGATGGTCTTGGTC.

### 2.6 Measurement of oxidative stress and inflammatory biomarkers

The activity of MDA in liver and GSSG and GSH in plasma were determined as performed in the kit. MDA was measured by thiobarbituric acid, and GSSG and GSH by dithiodinitrobenzoic acid (DTNB). MDA, GSSG, and GSH kits were purchased from Beyotime.

### 2.7 Immunohistochemistry (IHC)

Fixed tissue was cut into chips and rehydrated in a graded series of ethanol. The tissue chip was placed in the citrate buffer (pH = 6.0) for 40 min to repair the antigen, then incubated with 3% H_2_O_2_ for 25 min to inactivate the endogenous peroxidase and washed with PBS. Then, 3% BSA was added, and the chip was sealed for 30 min. After gently shaking off the solution, chips were incubated overnight with the antibody of anti‐ACE (24743‐1‐AP, Proteintech, China) or anti‐ACE2 (21115‐1‐AP, Proteintech, China). On the next day, the biotin‐conjugated AffiniPure goat anti‐rabbit IgG(H + L) (SA00004‐2, Proteintech, China) was added to the tissue chip and incubated at 28°C for 50 min. Finally, fresh DAB color‐developing solution was added. The software Aipathwell (Servicebio, China) was used to analyze the staining intensity and rate of the positive cells. The histochemistry score (H‐score) was used for each slide in order to evaluate the staining intensity. H‐Score (∑(pi×i) = (percentage of weak‐intensity cells ×1) + (percentage of moderate‐intensity cells ×2) + (percentage of strong‐intensity cells ×3), where i is the degree of positive cells: negative, no staining, 0 points; weakly positive, yellowish, 1 point; moderately positive, brownish, 2 points; strongly positive, brownish, 3 points; and pi is the percentage of positive cells of the corresponding degree. Then, we analyzed and calculated the grade, the measurement area, the positive area, the tissue area in the measurement area, the cumulative optical density (IOD), the mean optical density (MOD), and the positive area density (AD) (Zhao et al., 2022).

### 2.8 Data analysis and statistics

*n* = 6 in each group. All data were expressed as mean ± SD, a two‐tailed test was selected, and values of *p* < .05 were considered significant. GraphPad Prism 8.0 was used.

## 3 RESULTS

### 3.1 Wheat peptides and soy peptides reduce systolic blood pressure in rats

**FIGURE 1:**
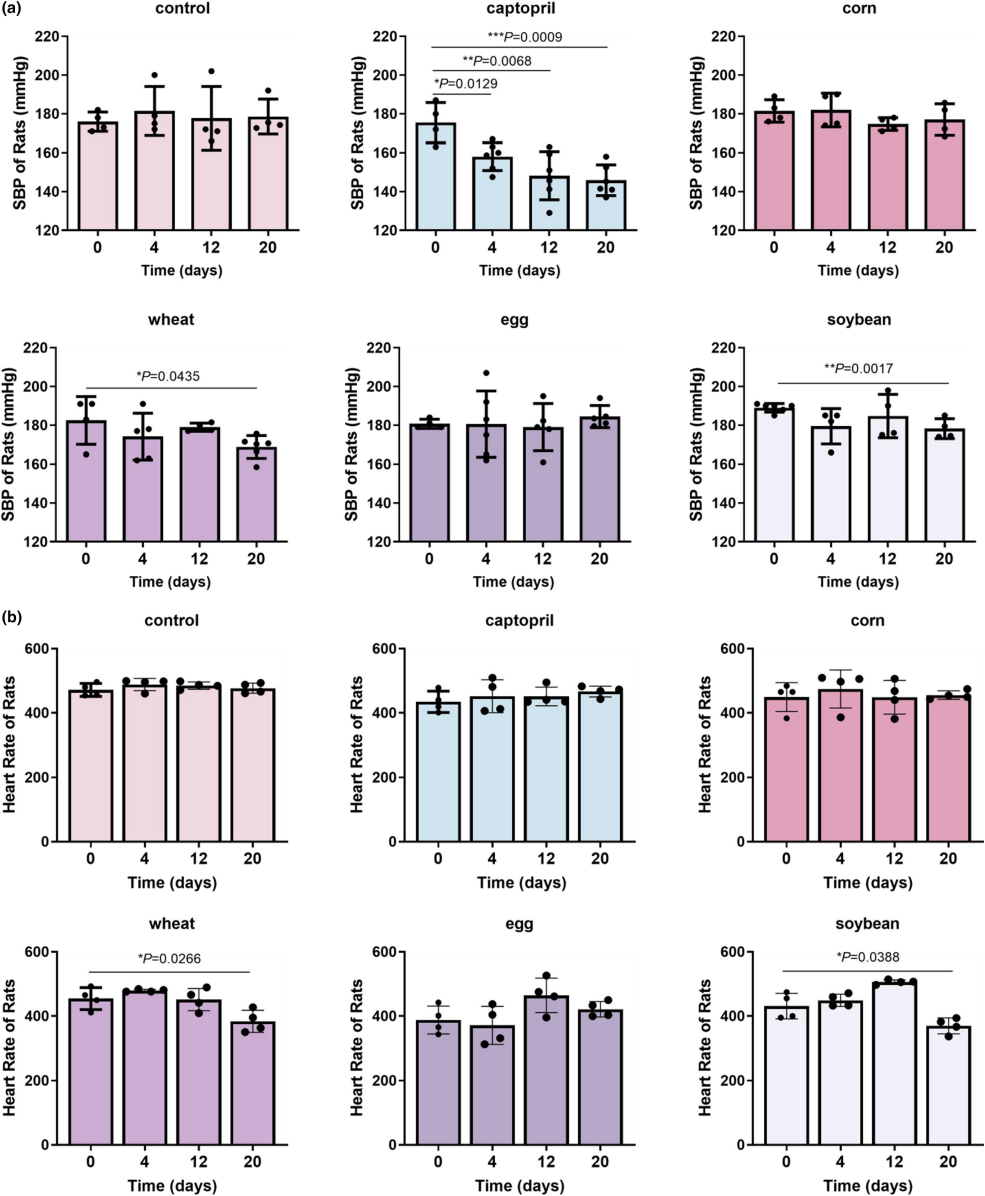
Peptide intervention has significant effect on blood pressure and heart rate in SHR. (a) Systolic blood pressure of SHR treated with high‐concentration peptides or captopril was measured for 20 days. (b) Heart rate of SHR treated with high‐concentration peptides or captopril was measured for 20 days. (Data represent means ± SE; *n* = 6; *p < .05; **p < .01; ***p < .001.)

According to the records, the use of peptides did not affect the growth of rats (Table 1). Systolic blood pressure (SBP) was in the range 170–190 mmHg in all groups before treatment, which met the criteria of a spontaneously hypertensive rat model. Captopril, an angiotensin‐converting enzyme inhibitor, has been used in the treatment of hypertension and this study was found to be a positive control. During the observation period, the SBP of the positive control group (158.03 ± 7.17 mmHg at day 4, *p* = .0129; 148.22 ± 12.43 mmHg at day 12, *p* = .0068; and 145.89 ± 7.91 mmHg at day 20, *p* = .0009) was always lower than that of the model control group (181.5 ± 12.66 mmHg at day 4, 177.75 ± 16.38 mmHg at day 12, and 178.63 ± 8.99 mmHg at day 20). After 20 days of intervention, SBP was significantly reduced in both groups of rats fed wheat peptide (from 182.5 ± 12.26 mmHg at day 0 to 168.86 ± 5.86 mmHg at day 20, *p* = .0435) and soybean peptide (from 189 ± 2.19 mmHg at day 0 to 178.25 ± 5.14 mmHg at day 20, *p* = .0017) ad libitum, whereas the change in systolic blood pressure was not significant in both groups of rats fed corn peptide (from 181.5 ± 5.8 mmHg at day 0 to 177.08 ± 8.11 mmHg at day 20, *p* = .4096) and egg white peptide (from 181.5 ± 5.8 mmHg at day 0 to 177.08 ± 8.11 mmHg at day 20, *p* = .4096) (Figure 1a and Table 2).

**TABLE 1:** Effects of different polypeptides on body weight.

Group name	Average body weight at day 0 (g)	Average body weight at day 20 (g)	Growth rate
CON	232.5	281.4	21%
CP	237.2	286.4	21%
WP	229.2	274.2	20%
EP	234.0	283.2	21%
SP	235.3	282.8	20%
Captopril	233.6	281.6	21%

*Note*: Data represent means; *n* = 6.

**TABLE 2:** Effects of different polypeptides on SBP.

Group name	SBP at day 0	SBP at day 20	*p*‐value
CON	176 ± 4.97	178.63 ± 8.99	0.237425939
CP	181.5 ± 5.8	177.08 ± 8.11	0.409579045
WP	182.5 ± 12.26	168.86 ± 5.86	0.043524955
EP	180.75 ± 2.36	184.5 ± 5.67	0.259413825
SP	189 ± 2.19	178.25 ± 5.14	0.001675594
Captopril	175.5 ± 10.34	145.89 ± 7.91	0.018666226

*Note*: Data represent means±SD; *n* = 6.

**FIGURE 2:**
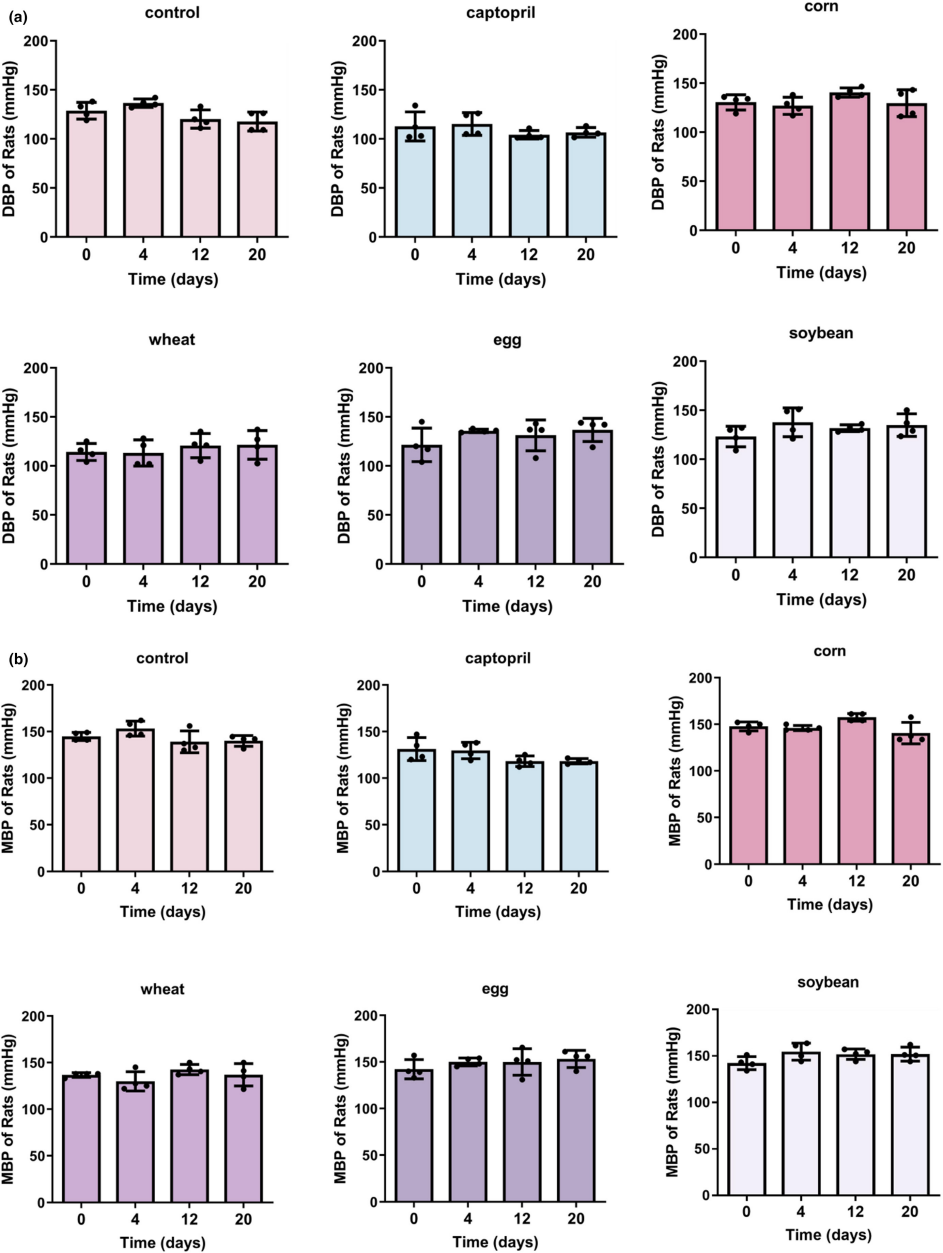
Peptide intervention changed unapparent on mean and diastolic blood pressure in SHR. (a) Mean blood pressure of SHR treated with peptides or captopril was measured for 20 days. (b) Diastolic blood pressure of SHR treated with peptides or captopril was measured for 20 days. (Data represent means ± SE; *n* = 6; *p < .05; **p < .01; ***p < .001.)

The heart rate data showed that the treatment of corn peptide and egg white peptide did not change the heart rate of rats. After 20 days of feeding wheat peptide (from 454.5 ± 34.02 mmHg at day 0 to 383.75 ± 34.48 mmHg at day 20, *p* = .0266) and soybean peptide (from 431 ± 39.51 mmHg at day 0 to 369.63 ± 24.7 mmHg at day 20, *p* = .0388), the heart rate of rats decreased significantly (Figure 1b), indicating that these two peptides have the effect of improving tachycardia in hypertensive rats. Treatment with captopril or other peptides did not affect diastolic and mean blood pressure in hypertensive rats (Figure 2a,b; Tables 3 and 4).

**TABLE 3:** Effects of different polypeptides on DBP.

Group name	DBP at day 0	DBP at day 20	*p*‐value
CON	128.75 ± 8.42	117.75 ± 9.72	0.137933027
CP	130.5 ± 7.77	129.58 ± 13.72	0.910903767
WP	114.25 ± 8.73	120.75 ± 12.28	0.42572905
EP	121.5 ± 17.14	136.75 ± 11.87	0.193776408
SP	123.25 ± 10.44	134.88 ± 11.51	0.185112632
Captopril	112.75 ± 14.86	106.67 ± 4.92	0.466489582

*Note*: Data represent means±SD; *n* = 6.

**TABLE 4:** Effects of different polypeptides on MBP.

Group name	MBP at day 0	MBP at day 20	P‐value
CON	144.75 ± 4.35	140.00 ± 5.79	0.237425939
CP	147.75 ± 4.79	140.59 ± 11.52	0.294404778
WP	136.75 ± 2.50	136.96 ± 12.00	0.974089623
EP	142.25 ± 10.31	153.25 ± 9.14	0.161435203
SP	142.25 ± 6.99	151.88 ± 7.47	0.108885381
Captopril	131.25 ± 12.34	118.21 ± 2.82	0.084948238

*Note*: Data represent means±SD; *n* = 6.

### 3.2 Wheat and soybean peptides downregulate ANG II by modulating angiotensin‐converting enzyme

**FIGURE 3:**
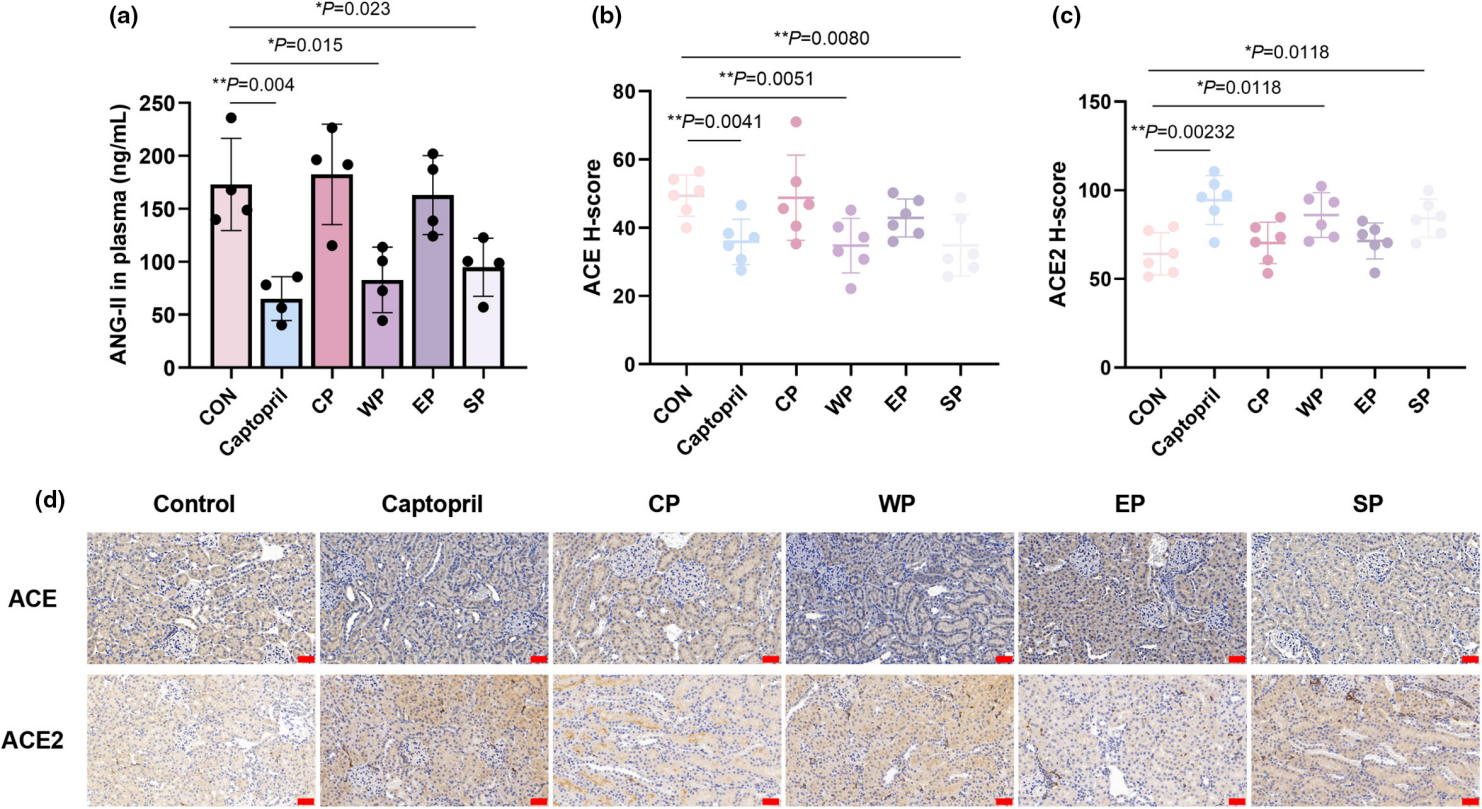
Wheat and soybean peptides downregulate ANG II by modulating angiotensin‐converting enzyme. (a) The concentration of angiotensin II (ANG II) in plasma detected using the rat ELISA kit. (b) Quantification of ACE expression in SHR kidney tissue using the H‐score. (c) Quantification of ACE2 expression in SHR kidney tissue using the H‐score. (d) Representative immunohistochemistry images showing ACE and ACE2 expression derived from kidneys of SHR as indicated (scale bars, 50 μm). (Data represent ± SE; *n* = 6; *p < .05; **p < .01; ***p < .001.)

To analyze the extent of hypertension in rats, we examined the classic molecular index angiotensin 2 (ANG II). After 20 days of peptide treatment, ANG II was significantly reduced in the group fed wheat and soy peptides compared to the control group fed normal water (Figure 3a), indicating that wheat and soybean peptides act on the renin‐angiotensin system to reduce blood pressure and improve vasoconstriction. To elucidate the mechanism of antihypertensive effects of food‐derived bioactive peptides, the IHC of angiotensin‐converting enzyme (ACE/ACE2) was added. As analyzed by IHC, ACE was significantly lower in the kidneys of the captopril, WP, and SP groups compared with the negative control group (Figure 3b,d). Angiotensin‐converting enzyme 2 (ACE2), an ACE homologous protein, effectively lowers blood pressure and vasodilates blood vessels through cleavage of ANG II. Consistent with the downward trend of ANG II, we observed that the protein level of ACE2 was significantly elevated in the captopril, WP, and SP groups (Figure 3c,d). The above data elucidate the molecular mechanism by which wheat and soybean peptides reduce blood pressure by modulating the protein levels of ACE and ACE2.

### 3.3 Wheat peptides enhance antioxidant capacity

**FIGURE 4:**
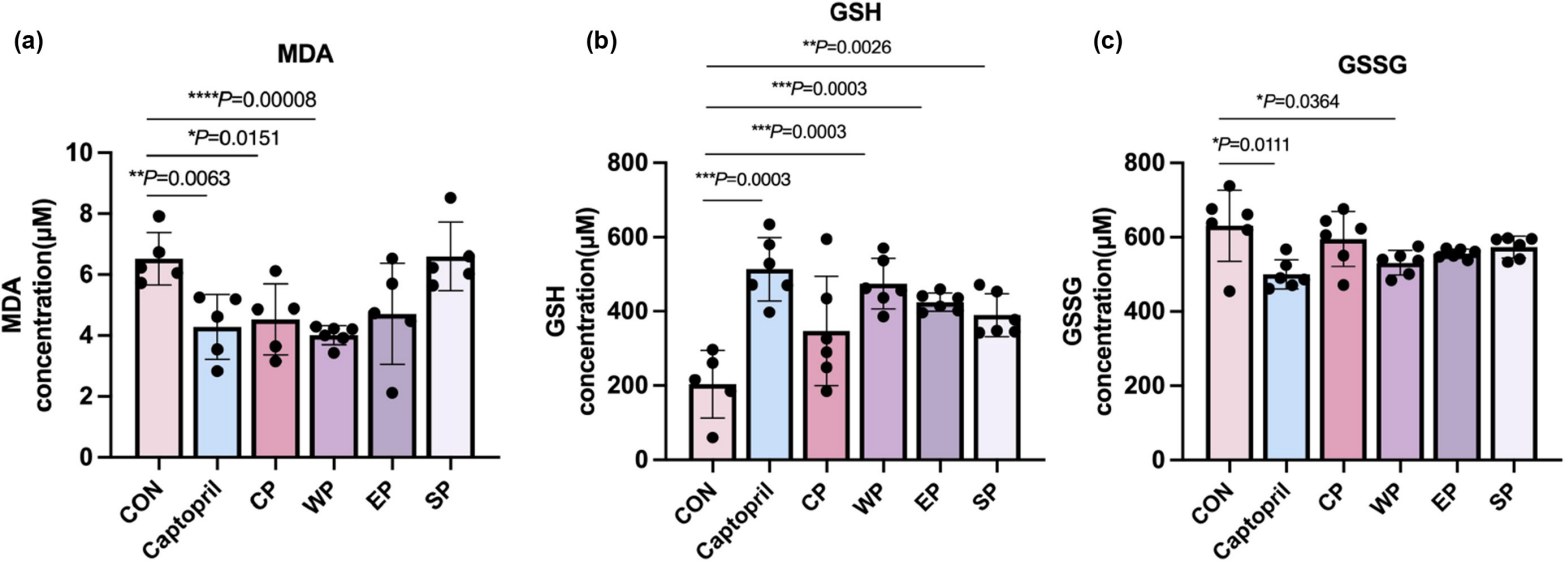
Wheat peptides enhance antioxidant capacity. (a) Effect of different peptides on MDA levels of liver in rats. (b) Effect of different peptides on GSH levels of plasma in rats. (c) Effect of different peptides on GSSG levels of plasma in rats (Data represent means ± SE; *n* = 6; *p < .05; **p < .01; ***p < .001.)

MDA in liver tissue was reduced significantly in the fed with wheat peptides (*p* = .00008) and corn peptides (*p* = .0151) (Figure 4a). To measure the antioxidant capacity, the effects of peptides on GSH content in rat liver are shown in Figure 4b. Compared with the control group fed with normal diet, the GSH was significantly increased in the polypeptide group, especially in the wheat peptides (*p* = .0003), which showed that wheat peptides significantly slowed down GSH consumption or promoted GSH synthesis in rats. The content of GSSG decreased differently in the peptide group and varied significantly in the WP group (*p* = .0364) (Figure 4c). This showed a consistent trend with GSH levels, which indicates that the peptide diet can prevent the decrease in GSH levels and GSSG levels in rats and protect cells from oxidative damage produced by free radicals. The results showed significant antioxidant effect of wheat peptides.

### 3.4 Wheat peptides affect the secretion of inflammatory factors

**FIGURE 5:**
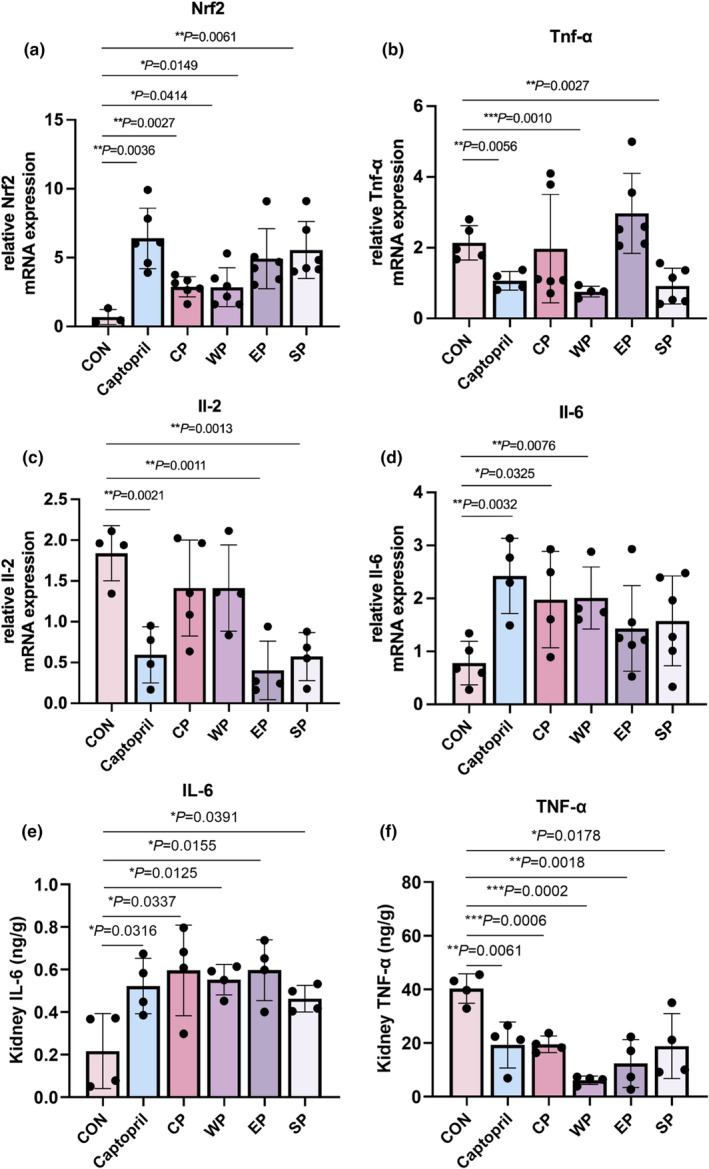
Effects of different polypeptides on inflammatory factors. (A–D) Measure the expression of *Nrf2*, *Tnf‐a*, *Il‐2*, and *Il‐6* in liver by RT‐qPCR. (E–F) Measure the protein level of IL‐6 and TNF‐α. (Data represent means ± SE; *n* = 6; *p < .05; **p < .01; ***p < .001.)

Hypertension shared a strong correlation with inflammation, so we further analyzed the effect of peptide treatment on the levels of inflammatory factors. Wheat peptide (*p* = .0414) could increase the Nrf2 mRNA expression while wheat peptide decreased the expression of *Tnf‐α* significantly (*p* = .0010) (Figure 5a,b). All four kinds of peptides upregulated the expression of *Il‐6* (*p* < .05) and *Il‐2* (*p* < .001) (Figure 5c,d). Using ELISA assay, it was found that the level of IL‐6 in rat kidneys was significantly increased compared with the control group with normal diet (Figure 5e), indicating that corn peptide, wheat peptide, egg peptide, and soybean peptide promoted renal inflammatory response after treatment for 20 days. TNF‐α inhibits the production of NO by endothelial cells, thereby adversely affecting vasodilation. The experimental data showed that the level of TNF‐α was significantly decreased after the treatment of corn peptide, wheat peptide, and egg peptide, and the level of TNF‐α in the wheat peptide treatment group was the lowest (Figure 5f), which was consistent with the significant reduction of blood pressure by the wheat peptide. There was a slight decrease in TNF‐α in the soybean peptide‐treated group, which may improve blood pressure by affecting other factors. In conclusion, wheat peptides affect the secretion of inflammatory factors.

## 4 DISCUSSION

The most obvious symptom of hypertension is that the blood pressure is higher than 140/90 mmHg. According to our data, the systolic blood pressure of rats in the control group with normal diet is always higher than 165 mmHg. With the long‐term continuous treatment of captopril, the systolic blood pressure of SHR gradually decreased and finally approached 140 mmHg, which indicated the function of antihypertension. Wheat peptide and soybean peptide could alleviate hypertension with a significant difference in the end, but the effect was weaker than captopril. Hypertension can damage cardiovascular function, one of the characteristics is that the heart rate is accelerated, and tachycardia can, in turn, increase the risk in hypertensive patients. The results showed that wheat peptide and soybean peptide alleviated tachycardia in rats with the agreement at the initial heart rate level of more than 400 beats/min. The renin–angiotensin–aldosterone system (RAAS) is a key hormonal system affecting hypertension. The abnormal increase in angiotensin can easily lead to renal hypertension, contraction of arteries, and upregulated blood pressure. Normally, the level of ANG II can reflect the degree of vascular tension in hypertensive patients. Continuous treatment of wheat and soybean peptides significantly downregulated the plasma ANG II level, which was consistent with the reduction in systolic blood pressure.

In hypertension, the transcription level of many cytokines related to inflammation in monocytes was significantly increased, and the total number of macrophages and dendritic cells was significantly increased (Loperena et al., 2018). It has been documented that hypertension is associated with the accumulation of T cells and monocytes/macrophages in the blood vessels and kidneys that produce potent cytokines that affect vascular and renal function (McMaster et al., 2015). Nitric oxide (NO) is a key molecule that regulates vasodilation. TNF‐α has many adverse effects, one of which is leading to hypertension. Through the analysis, it was found that wheat peptides had the effect of reducing TNF‐α, which may achieve the effect of dilating blood vessels by promoting the expression of NO. In addition, TNF‐α can also promote the reabsorption of sodium in renal medulla by interfering with tubulovascular crosstalk, resulting in renal sodium retention (Lu & Crowley, 2018). It is suggested that wheat peptides may have the effect of alleviating renal sodium retention. IL‐6 is associated with blood pressure in hypertensive subjects and is reduced by treatment with an ANG II receptor blocker (Vazquez‐Oliva et al., 2005). Various peptides upregulated IL‐6 in the kidney, but the background levels were less than 1 ng/g, indicating that the continuous stimulation of peptides caused a slight upregulation of IL‐6, resulting in a slight inflammation in the kidney tissue, but did not affect arterial blood pressure.

MDA content can reflect the speed and intensity of body peroxidation, which is used as an index to evaluate lipid peroxidation damage (Haberland et al., 1990). Compared with the CON group, rats had no difference between EP and SP groups, indicating that egg white and soybean peptides had little effect on lipid oxidative damage in the animals. However, the MDA content of CP and WP rats was significantly different. Corn and wheat peptides can reduce the accumulation of MDA in rat livers, indicating that corn peptides and wheat peptides can enhance antioxidant enzyme activity, effectively remove excess ROS, and reduce oxidative stress response, which could antiaging. In the peptide group, the GSH content in the CP, EP, and SP groups have no significant difference compared with the CON group, while the WP group was significantly increased, probably because the wheat peptide activated the GSH synthesis pathway or provided raw material for the synthesis of GSH. Therefore, wheat polypeptides can alleviate or even improve the reduction in GSH content in animals, increase the content of antioxidant factors in the body, and improve the antioxidant capacity.

The core molecule of Nrf2‐ARE signaling pathway, Nrf2, is located in the cytoplasm, and normally is inactive (He et al., 2020). Under the stimulation of wheat peptide, the expression of Nrf2 was increased significantly. If Nrf2 activated transport into the nucleus, it can combine with ARE to activate the expression of target genes, regulate the transcription of antioxidant enzymes and proteins, protect the body from reactive oxygen and other substances, and inhibit the cell aging caused by oxidative stress (Ruiz et al., 2013). Oxidative and inflammatory reactions are accompanied by the whole life process of the body, and the accumulation of excessive oxidative stress and inflammatory reactions is also an important cause of aging, and effective confrontation can delay the aging process.

## 5 CONCLUSION

After 20 days of free feeding, the wheat peptide and the soybean peptide have the effect of reducing systolic blood pressure of SHR. These two peptides are proven to relieve the symptoms of hypertension under long‐term administration with high concentration. Considering the results of the viability of antioxidant enzymes and the expression of inflammatory factors, we provide an experimental basis for the development of natural peptides as protective agents against antioxidants in hypertension animals.

## AUTHOR CONTRIBUTIONS

Yuefan Zhou and Yixin Xu contributed to project development, data collection, data analysis, and writing. Tongguan Tian performed the experiments. Yanping Xu conceived the project, designed the experiments, and wrote the paper.

## FUNDING INFORMATION

Supported by funding from the Nourse Centre for Pet Nutrition.

## CONFLICT OF INTEREST STATEMENT

The authors declare that they have no known competing financial interests or personal relationships that could have appeared to influence the work reported in this paper.

## ETHICS STATEMENT

This study was approved by the Institutional Review Board of Tongji University and received ethical approval with the number TJ‐HB‐LAC‐2023‐48. Animal experiments were in accordance with the principles of animal welfare.

## Data Availability

The data that support the findings of this study are available from the corresponding author upon reasonable request.
